# Changes in Cyclic Guanosine Monophosphate Channel of 661w Cells In vitro with Excessive Light Time

**DOI:** 10.18502/jovr.v18i4.14554

**Published:** 2023-11-30

**Authors:** Yahan Zhang, Rui Yin, Xin Liu

**Affiliations:** ^1^Department of Ophthalmology, Shanghai General Hospital, Shanghai Jiao Tong University, National Clinical Research Center for Eye Diseases, Shanghai Clinical Research Center for Eye Diseases, Shanghai Key Laboratory of Ocular Fundus Diseases, Shanghai Engineering Center for Precise Diagnosis and Treatment of Eye Diseases, Shanghai, China; ^2^Department of Biomedical Informatics, Harvard Medical School, Boston, MA, USA; ^3^Department of Ophthalmology, The Second Hospital of Jilin University, Changchun, China

**Keywords:** Retinal Light-induced Injury, cGMP-gated Channel, Photoreceptor Cells

## Abstract

**Purpose:**

To determine the response time and protective mechanism of the cyclic guanosine monophosphate (cGMP) channel in 661w cells.

**Methods:**

661w cells were exposed to 4500Lux visible light for three and four days at the following exposure time periods per day: 20, 30, 60, 90, 120, and 180. Cells were incubated for the rest of the time without any other treatment. Cell activity and cell death rates were measured with Hoechst/PI (diphenylmethane/propidium iodide) staining. Western Blot was used to detect the levels of guanylate cyclase-activating proteins 1 (GCAP1), cGMP, and phosphodiesterase (PDE)6 in the cGMP-gated channel.

**Results:**

661w cells showed low mortality within three days. The mortality rate increased from the fourth day, especially during the longer times (120 and 180 min) of light exposure. After three-day illumination, the level of cGMP increased after 20 and 90 min and the level of GCAP1 increased after 60 and 90 min. After four days of illumination, the level of GCAP1 upregulated after a time of 20 and 60 min, while the cGMP level decreased from 30 min. The expression of PDE6 upregulated at each light period.

**Conclusion:**

The survival rate of 661w cells was relevant to the time of light exposure. The changes in GCAP1, cGMP, and PDE6 levels over time were possibly related to cell metabolism and restoration after light-induced damage.

##  INTRODUCTION

Visible light is essential for inducing vision and regulating the circadian rhythm. Prolonged exposure to intensive light is harmful to the retina^[1]^ and can lead to severe retinal degeneration, including age-related macular degeneration (AMD) and retinitis pigmentosa (RP), resulting in poor vision or irreversible blindness.^[2]^ Additionally, excessive light has adverse effects on retinal photoreceptor cells, retinal pigment epithelial (RPE) cells, and retinal ganglion cells (RGC).^[3]^ Retinal photoreceptors are the key cells in visual phototransduction, which are located in the outer layer of the retina and transmit light stimuli from the optic nerve to the brain.^[4]^ After light exposure, photoreceptor cells are initially damaged at the distal end of the rod out segment (ROS) which progresses over time to the entire ROS.^[5]^ It has been reported that peroxide reactivity in the ROS increased after intense light exposure.^[6]^ However, the underlying molecular mechanism of retinal light damage is unclear, and effective protection is lacking in the clinic.

**Figure 1:**
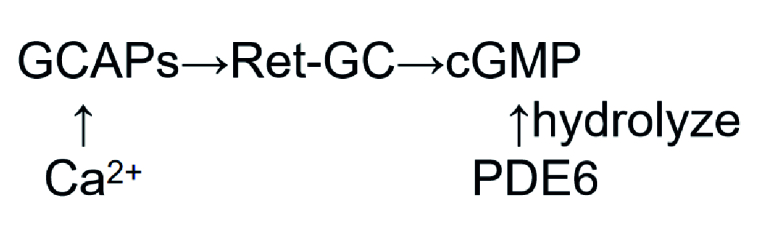
Illustrates the response chain of the cGMP-gated channel. In the light, the concentration of Ca ${}^{2+}$ is decreased and cGMP is hydrolyzed by PDE6, which activates the cGMP-gated channel to produce cGMP by adjusting GCAPs and Ret-GC proteins.

The cyclic guanosine monophosphate (cGMP) channel is closely related to the visual excitation of photoreceptors and the enzymatic cascade.^[7]^ In the dark, a high concentration of cGMP from the cGMP channel keeps sodium channels open in the outer segment,^[8]^ while the level of cGMP decreases due to the activation of phosphodiesterase 6 (PDE6) in the light.^[9]^ cGMP channels control the activation of an enzymatic cascade [Figure 1] where photoreceptors absorb the photon and activate hydrolysis of cGMP by PDE6. The decreased concentration of cGMP followed by the closure of the cGMP-gated channel and a decrease in calcium ions (Ca ${}^{2+)}$ concentration, induces retinal guanylate cyclase (Ret-GC) via guanylate cyclase activating proteins (GCAPs)^[10]^ to produce cGMP. The feedback catalyzes the resynthesis of cGMP and releases free Ca ${}^{2+}$ ,^[11]^ which allows the photoreceptors to gradually recover the light sensitivity between darkness and light. However, prolonged exposure to large amounts of light could interrupt the balance of the cGMP-gated channel and in turn trigger cell death.^[1]^

661w cells were cloned from retinal tumors of a transgenic mouse line with the characteristics of cone photoreceptor cells. 661w cells are also sensitive to photooxidative stress, and they are used as an *in vitro* model to study cone photoreceptor cell biology and relevant diseases.^[12]^ Under light conditions, the cGMP channel in cone cells is activated as an internal transmitter to balance the concentration of Ca ${}^{2+}$ and proteins.^[13]^ In a study by Imanishi et al, immunohistochemistry and *in situ* hybridization was done in mouse and human retinas, they demonstrated that cone photoreceptor cells express relatively high levels of Ret-GC1 and GCAP1.^[14]^

Prolonged exposure to light decreased the sensitivity to light and caused light-induced damage. cGMP-gated channel triggered cell death. Therefore, as the reduction of light damage is of great significance, we investigated the response time and protective mechanism of the cGMP-gated channel and the associated triggered cell death in retinal photoreceptor cells.

##  METHODS

### Reagents and Materials

Invitrogen (Beijing, China) provided the cell culture medium and additives, while DingGuo BioTech (Beijing, China) supplied the plastic petri dishes. The mouse β-actin monoclonal antibody (#21800) was ordered from the SAB Biosciences Company (Maryland, USA). The rat cGMP monoclonal antibody (sc-390695), mouse GCAP1 monoclonal antibody (sc-136313), and mouse PDE6β monoclonal antibody (sc-377486) were all supplied by the SANTA Biotech Company (California, USA). Hoechst No.33342 was also from DingGuo Biotech, and propidium iodide (PI) was from the Cell Signaling Technology Company (Massachusetts, USA). The Endotoxin-Free Plasmid Mini-Preps Kit (B518161) was from Sangon Biotech (Shanghai, China).

### Cell Culture

661w cells were grown in a modified medium (DEME; Invitrogen, Beijing, China) supplemented with 100U/ml penicillin, 10% heat-inactivated fetal bovine serum, and 100 mg/ml streptomycin in an incubator of 95% air and 5% CO ${}_{2}$ at 37 ${}^{\circ }𝙲$ . 661W cells were generally passaged by trypsin at a ratio of one to six every three to four days.

### Visual Light Exposure

The 661w cells were exposed to 4500Lux visible light, which excluded wavelengths below 400 nm and above 800 nm. The light source was placed about 20 cm directly over the 6- or 96-well cell culture plates. To control the darkness, a lucifuge box was placed in the same incubator to create a dark chamber. Thus, cells were maintained in the same selected culture conditions, except during light exposure time. The medium under the dark and light conditions was checked over a one- to four-day period.

In addition, we set the periods as (three and four days), and cells were exposed to light for 20, 30, 60, 90, 120, and 180 min per day.

**Table 1:** The arrangement of exposure time.


orange**Light time (day)**	orange**0 min/d**	orange**20 min/d**	orange**30 min/d**	orange**60 min/d**	orange**90 min/d**	orange**120 min/d**	orange**180 min/d**
3 days	control 1	test 1	test 2	test 3	test 4	test 5	test 6
4 days	control 2	test 7	test 8	test 9	test 10	test 11	test 12
	
	

### Hoechst/ PI Staining

The 661w cells were first cultured in a 96-well plate for 24 hr. After being exposed to light, the cells were added to Hoechst solution, which was diluted using 1µg/ml phosphate-buffered saline (PBS) and incubated for 10 min at 37 ${}^{\circ }𝙲$ . The cells were then treated with the propidium iodide (PI) solution at a final concentration of 2 µg/ml and incubated in the dark for 15 min at 4 ${}^{\circ }𝙲$ . The Hoechst/PI-positive cells were observed by using an inverted fluorescence microscope.

### Western Blotting

After culturing in either light or dark, the 661w cells were collected and shaken in protein lysate buffer (20 mM Tris-HCl, PH 7.4, 25 ${}^{\circ }𝙲$ , 2 mM EDTA, 0.5 mM ethyl glycol tetraacetic acid [EGTA], 1 mM dithiothreitol, 50 mg/ml leupeptin, 50 mg/ml pepstatin A, 50 mg/ml aprotinin, and 0.1 mM phenylmethylsulphonyl fluoride). We used the bicinchoninic acid assay to check the protein level.^[16,27]^ An equal amount (20 µg) of cell lysate was dissolved in the sample buffer (62.5 mM Tris-HCl, pH 7.4,4% sodium dodecyl sulfate, 10% glycerol, 10% β-mercaptoethanol, and 0.002% bromophenol blue). The samples were then boiled for 3 min. Electrophoresis was utilized with 10% polyacrylamide gels containing 0.1% sodium dodecyl sulfate.^[17]^ We transferred the proteins to nitrocellulose membranes and incubated them for 3 hr at room temperature with primary antibodies. Then the proteins were incubated with the appropriate secondary antibodies. The signals were detected using the enhanced chemiluminescence Western Blotting detection reagent (Amersham Biosciences, USA) and exposed to an X-ray film. Densitometry analysis was performed with Quantity One software (Bio-Rad laboratories).

### Statistical Analysis

Each experiment was repeated at least three times. Data were shown as mean ± SEM. One-way analysis of variance (ANOVA) test was used for comparison of the above three groups. *P* < 0.05 was considered to be statistically significant.

The study complied with the World Medical Association Declaration of Helsinki, and the study protocol was reviewed and approved by the Ethical Committee (the tab of animal experimental ethical inspection, Jilin University, China, KT202003190).

##  RESULTS

**1.** **The mortality rate of 661W cells after being exposed to light.**

**Figure 2:**
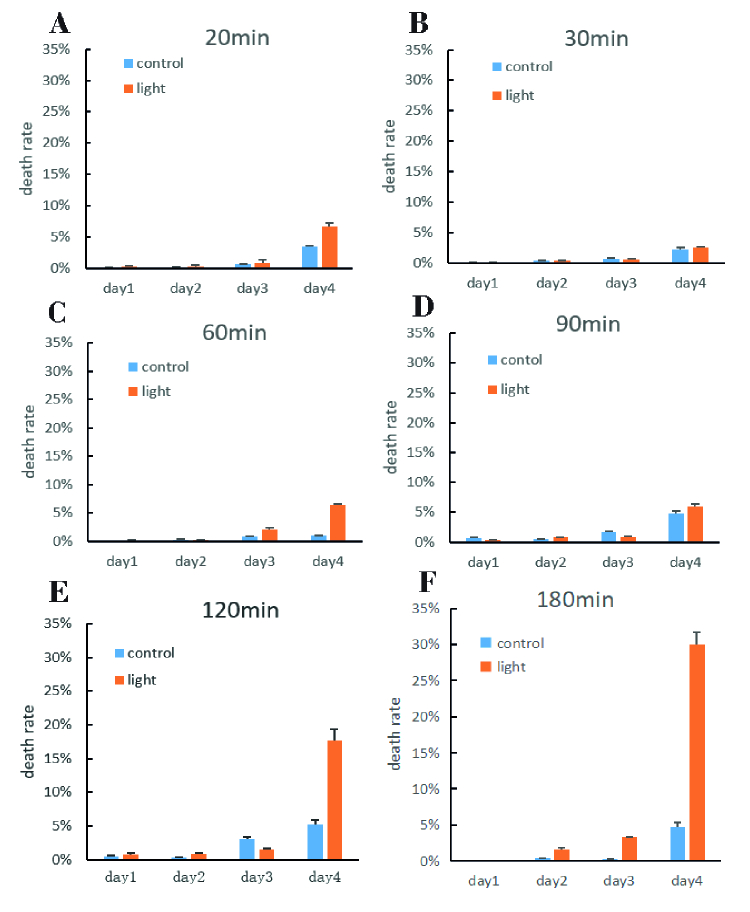
Comparison of the death rate between the dark and the light time in each time period. The cells in the control group were in the dark. The death rate was measured by Hoechst/PI staining. PI-positive cells were classed as dead cells. Death rate = PI positive cells/all cells (%). The mortality rate of 661w cells was below 5% in three days, while the mortality rate was above 5% after four days, and even reached 20% after 120 min and 30% after 180 min, *n* = 3.

The mortality rate of the 661W cells in each light time period (0, 20, 30, 60, 90, 120, 180) was low within three days. Only a few amounts of dead cells could be detected via red fluorescence with PI staining. From the fourth day, the cell death rate increased above 5% [Figure 2]. The mortality rate up-surged according to the length of exposure time: the longer the exposure time, the higher the death rate.

**2.** **The relationship between cGMP-gated channel and light exposure time in 661w cells.**

**Figure 3:**
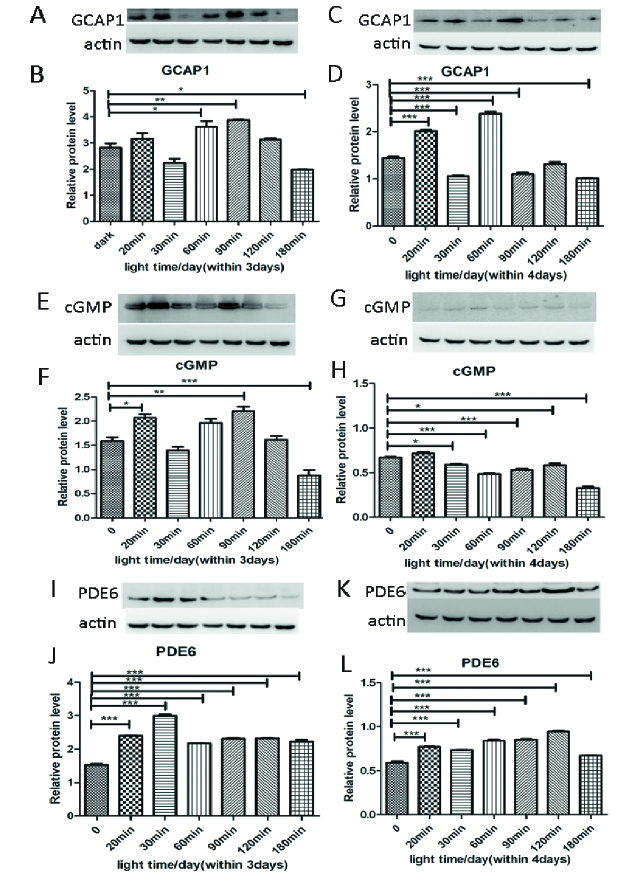
The expressions of GCAP1, cGMP, and PDE6 with the exposure time was measured by Western Blot. After three-day illumination, GCAP1 level increased significantly after 60 and 90 min, and cGMP level increased after 20 and 90 min. The level of PDE6 increased from 20 min. After four-day illumination, the GCAP1 level increased after 20 and 60 min but decreased after 30 and 90 min. The level of cGMP decreased after 30 min, while the level of PDE6 gradually increased from 20 min. Data were presented as mean ± SEM and one-way ANOVA (*n* = 3).

The western blot showed that the level of GCAP1 increased after exposing for 60 and 90 min for three days [Figure 3B]; the level of cGMP significantly increased after 20 and 90 min [Figure 3F]; and the level of PDE6 increased continuously from an exposure time of 20 min [Figure 3J]. However, the expression of GCAP1 and cGMP was much lower after a period of 180 min of light exposure. After being exposed to light for four days, the GCAP1 level increased after an exposure time of 20 and 60 min and decreased after 30 and 90 min [Figure 3D], while the level of cGMP decreased from 30 min [Figure 3H]. The expression of GCAP1 and cGMP significantly decreased after 180 min [Figure 3C & 3G]. The level of PDE6 gradually increased in pace with the prolonged exposure time [Figure 3L]. Thus, light damage of retinal photoreceptor cells was minor within three days, and 661w cells activated cGMP-signaling to balance the light condition, then repaired the function of the photoreceptor cells gradually from the fourth day.

##  DISCUSSION

The cGMP-gated channel is one of the critical molecular channels that affects the response to light and is associated with retinal degeneration. In the cGMP-gated channel, GCAP1 is the first signal protein that activates RetGC1 in the response, and then RetGC1 regulates the synthesis of cGMP to maintain the function of photoreceptors.^[18,20]^ Exposure to intensive light for a long time is a threat to 661w cells.

Prolonged light exposure caused an increased mortality rate of 661w cells. Cells were stable within three days; however, the death rate of cells surged after the fourth day with 120 or 180 min of light exposure. Chen et al reported that 661w cells produced nearly two-fold reactive oxygen species while ATP content decreased by 50% after blue LED light illumination, which reflected cell viability and death rate.^[19]^ Thus, the mortality rate of 661w cells increased in a time-dependent fashion after being exposed to light. In our study, exposure for 20, 30, and 60 min within three days was the safe period. The 661w cells could activate the self-regulation system to adjust to the extensive light exposure within three days, but lost this ability after four days. Additionally, the increased death rate of 661w cells occurred in periods of 120 and 180 min after four days. Therefore, the activation of the self-protection system of the 661w cells relied on the shorter length of light exposure time.

We supposed that the self-regulation was relevant to the cGMP-gated channel. After the 661w cells were exposed to light for three days, the level of cGMP increased after 20 and 90 min and the level of GCAP1 expressed after 60 and 90 min. However, the expression of these two proteins decreased after 180 min. PDE6 continuously expressed in the light, especially after 20 and 30 min. Our results suggested that the cGMP-gated channel was first stimulated after 20 min, recovered at 90 min, and disordered at 180 min within three days. After light illumination, the level of GCAP1 was gradually activated, simultaneously leading to the synthesis of cGMP.^[28]^ A high level of cGMP was found after 20 min, and then the expression of cGMP began to decline due to being hydrolyzed by PDE6.^[21,22]^ The level of GCAP1 was first highly increased after 60 min and then began to decrease. The expression of cGMP and GCAP1 increased again after the 90-min time period but decreased with prolonged time. This fluctuation might be the feedback of self-protection activated within the 661w cells. PDE6 continued to hydrolyze cGMP. Nevertheless, the levels of cGMP and GCAP1 decreased from 90 min and expressed lowest after 180 min, which indicated the imbalance of the cGMP-gated channel. Thus, the serious light-induced injury of the 661w cells was initiated from 180 min within three days. Furthermore, the expression of cGMP decreased from the 30-min time period after exposure to light for four days. The level of GCAP1 increased after 20 and 60 min but decreased after 30 and 90 min. Hence the cGMP-gated channel activated around 30 min to balance the proteins after four-day illumination. In addition, the level of PDE6 was always expressed constantly because of the hydrolysis function of PDE6 induced by light.^[23,30]^ However, the expression of GCAP1 and cGMP did not increase after 60 min. After being exposed to light for four days, we proposed that the cGMP-gated channel could not maintain the ability to regulate light conditions after 60 min. The mortality rate of 661w cells also increased after four days. Therefore, the protection of the cGMP-gated channel in 661w cells was disturbed by light after 60 min exposure for four days.

In summary, we demonstrated that the expression of proteins in the cGMP-gated channel was dependent on the light exposure time. The safe exposure time of 661w cells was < 90 min each day within three days or < 60 min each day within four days because cells could initiate the self-protection of the cGMP-gated channel. Photoreceptor cells might become unstable because of light-induced injury leading to retinal degenerative diseases.^[24,29]^ Until now, we were aware that natural and synthetic antioxidants and neuroprotective factors could reduce or prevent retinal light damage.^[25,26]^ Furthermore, we could explore the treatment depending on the cGMP-gated channel to protect the retina.

##  Financial Support and Sponsorship

None.

##  Conflicts of Interest

None.
